# Effects of different dual-task trainings on gait and cortical activation during obstacle crossing in stroke patients: a randomized controlled trial

**DOI:** 10.3389/fphys.2026.1869914

**Published:** 2026-06-18

**Authors:** Xianglin Wan, Zihao Zhu, Feng Xu, Qiujie Li

**Affiliations:** 1 School of Sport Science, Beijing Sport University, Beijing, China; 2 Key Laboratory for Performance Training & Recovery of General Administration of Sport of China, Beijing Sport University, Beijing, China; 3 Department of Rehabilitation, People’s Hospital of Queshan, Zhumadian, China; 4 China Institute of Sport and Health Science, Beijing Sport University, Beijing, China

**Keywords:** cerebral cortex, dual-task training, functional near-infrared spectroscopy, gait, stroke rehabilitation

## Abstract

**Objective:**

After stroke, patients often exhibit abnormal gait and cortical activation patterns during obstacle crossing, which heightens their risk of falls. This study aimed to examine the effects of Motor dual-task gait training (MDTG) and cognitive dual-task gait training (CDTG) on gait and cortical activation during obstacle crossing in stroke patients, to clarify their differential effects and underlying neural mechanisms.

**Methods:**

35 stroke patients were recruited and randomly assigned to either the MDTG group (*n* = 17) or the CDTG group (*n* = 18), and were assigned to receive the corresponding intervention training. Participants in both groups underwent a 4 week training program, consisting of three sessions per week. Spatio-temporal parameters, lower limb joint angles and moments, and changes in oxyhemoglobin concentrations (ΔHbO_2_) were assessed in patients during obstacle crossing both pre- and post-training.

**Results:**

Following training, both groups demonstrated significant improvements in stride length, step length, step width, gait speed, toe-obstacle clearance of the leading and trailing limb, toe-obstacle distance of the trailing limb, anteroposterior and mediolateral COM displacement, mediolateral COM mean velocity, anteroposterior COM-COP distance, hip flexion angle of the leading limb, knee flexion angle of the trailing limb, and peak hip flexion moment of the trailing limb (*P* < 0.05). Concurrently, following the training, ΔHbO_2_ in the bilateral prefrontal cortex was significantly reduced during obstacle crossing (*P* < 0.05). However, no significant differences were observed between the two groups for any parameters (*P*>0.05).

**Conclusion:**

A 4-week program of either MDTG or CDTG effectively improved postural stability, enhanced control of the swing limb and COM, and increased efficiency in cortical resource utilization during obstacle crossing in stroke patients. However, no differences in these effects were observed between the two training modalities.

**Clinical Trial Registration:**

https://www.chictr.org.cn/showproj.html?proj=200755, identifier ChiCTR2300077304.

## 1 Introduction

Falls are among the most common complications after stroke and are frequently accompanied by fractures, depression, and other adverse outcomes, severely compromising patients’ quality of life and slowing their rehabilitation progress (Pouwels et al., 2009; Schmid et al., 2009). Obstacle crossing is the leading scenario for falls in stroke patients, accounting for approximately 33% of all fall events (Wan et al., 2025). Our recent prospective and retrospective studies (Wan et al., 2025; Zhu et al., 2025) have shown that neuromuscular impairments such as lower-limb weakness, spasticity and proprioceptive deficits commonly lead patients to exhibit abnormal gait patterns during obstacle crossing. These patterns are characterized by reduced toe-obstacle clearance and heel-obstacle distance, in addition to insufficient ankle plantarflexion moment of the stance limb (Wan et al., 2025; Zhu et al., 2025). Such gait characteristics are closely associated with an elevated risk of falls (Wan et al., 2025; Zhu et al., 2025). Therefore, developing scientific rehabilitation strategies to improve gait performance during obstacle crossing in stroke patients is critical for reducing fall risk and enhancing their independent mobility and quality of life.

Dual-task gait training has been shown to effectively improve gait performance and is widely used in the rehabilitation of stroke patients (Yang et al., 2007; Subramaniam et al., 2014; Zhang et al., 2022). Depending on the training content, it can be classified as motor dual-task gait training (MDTG) and cognitive dual-task gait training (CDTG) (Her et al., 2011). MDTG superimposes a concurrent motor task on walking to challenge motor control and postural regulation under dual motor loads, whereas CDTG introduces a concurrent cognitive task to impose cognitive interference, thereby placing greater demands on attentional allocation and executive control. Despite these differences in task structure, both paradigms enhance attentional switching and refine the allocation of cognitive resources in stroke patients (Zhang et al., 2022; Liu et al., 2024). Existing studies (Kim et al., 2016; Liu et al., 2017; Kim and Kim, 2018; Iqbal et al., 2020; Zhang et al., 2022; Huimeng et al., 2025) have shown that dual-task training can improve gait speed, balance, lower-limb coordination, and task performance in patients during single-task or dual-task walking. However, the differential effects of the two types of dual-task training on gait improvement have not been sufficiently compared. In addition, in high-risk scenarios such as obstacle crossing, the effects of the two types of dual-task training on gait parameters in stroke patients remain unclear, and the differential effects of these trainings require further investigation.

The mechanisms by which dual-task gait training improves walking ability in stroke patients may be closely related to the modulation of neuroplasticity. Evidence from functional near-infrared spectroscopy (fNIRS) studies has shown that stroke patients exhibit increased prefrontal cortex (PFC) activation during complex walking tasks such as obstacle crossing, which may reflect a high demand on executive control resources and a compensatory reliance on cortical resources (e.g., attentional allocation and motor planning), thereby limiting their ability to adapt to complex environments. Relevant intervention studies have demonstrated that CDTG can modulate the PFC activation patterns during walking under cognitive dual-task conditions and enhance functional network connectivity across brain regions (Collett et al., 2021; Xie et al., 2024). However, longitudinal evidence regarding the effects of dual-task training on cortical activation patterns during obstacle crossing in stroke patients remains limited, particularly for MDTG, where fNIRS evidence is still scarce. Moreover, it remains unclear whether different types of dual-task training differentially influence cortical activation patterns during obstacle crossing. Changes in cerebral oxygenation monitored by fNIRS can indirectly reflect adjustments in cortical activation patterns, offering valuable insight into the neural mechanisms by which dual-task training facilitates rehabilitation.

Therefore, this study aimed to investigate the effects of CDTG and MDTG on gait and cortical activation patterns during obstacle crossing in stroke patients, with the goal of clarifying the differential effects of these training modalities and their underlying neural mechanisms, thereby providing a theoretical basis for optimizing rehabilitation strategies. We hypothesized that: (1) both types of dual-task gait training would improve gait characteristics during obstacle crossing in stroke patients, manifested as increased gait speed, longer step length and limb-obstacle distance, greater lower limb joint angles and moments, and increased center of mass (COM) velocity and displacement; (2) both types of dual-task gait training would enhance the efficiency of brain resource utilization during obstacle crossing in stroke patients, as reflected by reduced PFC activation and premotor cortex (PMC) activation; (3) compared with MDTG, CDTG would produce greater improvements in gait and cortical activation patterns during obstacle crossing in stroke patients.

## 2 Methods

### 2.1 Participants

#### 2.1.1 Inclusion criteria

(1) diagnosis of cerebral hemorrhage or cerebral infarction confirmed by computed tomography (CT) or magnetic resonance imaging (MRI); (2) unilateral hemiparesis; (3) being in a non-acute stage with stable vital signs; (4) age between 40 and 65 years; (5) a Berg Balance Scale (BBS) score > 45; (6) a Mini-Mental State Examination (MMSE) score > 24; (7) ability to understand and follow therapists’ instructions; (8) ability to walk independently without orthosis and/or assistance from others; (9) ability to lead with the affected limb to independently cross the 10% leg-length obstacle.

#### 2.1.2 Exclusion criteria

(1) comorbidities, disabilities, or other neurological or orthopedic disorders, apart from stroke, that could affect gait training; (2) severe hearing or visual impairment, or aphasia that precludes completion of training; (3) contraindications to exercise or other uncontrollable factors; (4) participation in other training programs during the study period.

**Figure 1:**
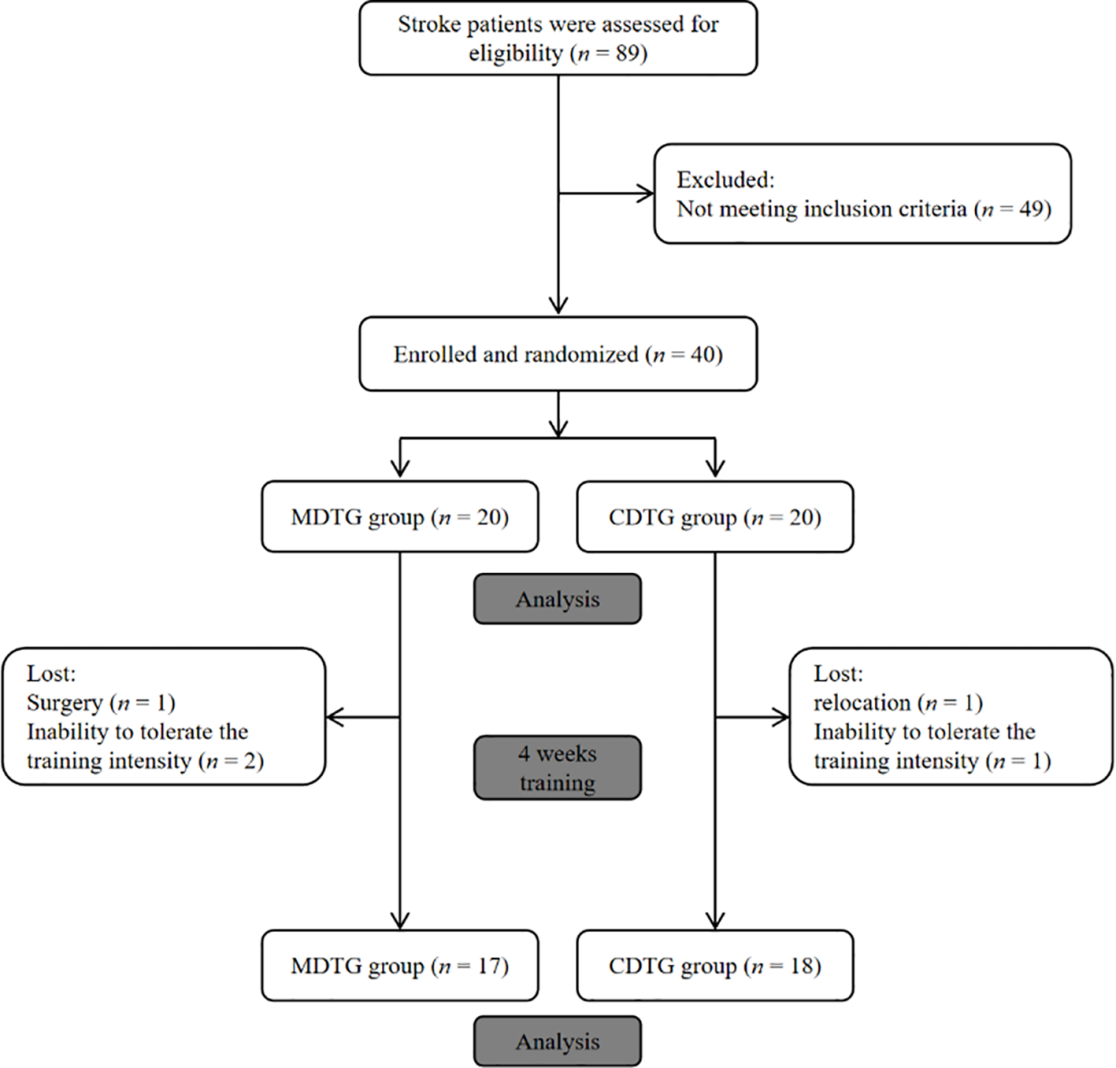
CONSORT flow diagram. MDTG denotes the motor dual-task gait training; CDTG denotes the cognitive dual-task gait training.

Sample size estimation was performed using G*Power 3.1 for a two-way repeated measures ANOVA (within-between interaction). Based on a previous study of similar design (Chen et al., 2023), the significance level (α) was set at 0.05, statistical power at 95%, the effect size (Cohen’s *f*) at 0.5 (medium effect), the correlation among repeated measures at 0.5, and the nonsphericity correction (*ϵ*) at 1. The calculation indicated that at least eight participants were required per group, yielding a total sample size of 16. Considering the potential for participant dropout in interventional studies, a total of 40 stroke patients admitted to the Department of Rehabilitation at People’s Hospital of Queshan were ultimately recruited according to the inclusion and exclusion criteria. Participants were randomly assigned to either the MDTG group or the CDTG group using a random number table generated by an independent researcher not involved in the assessment or intervention procedures. Outcome assessments and data analyses were conducted by assessors blinded to group allocation. During the course of the study, five participants withdrew due to surgery, relocation, or inability to tolerate the training intensity, leaving a total of 35 participants who completed the study, with 17 in the MDTG group and 18 in the CDTG group (**Figure 1**). There were no statistically significant differences in demographic characteristics between the two groups (*P* > 0.05) (**Table 1**).

**Table 1:** Demographic characteristics of participants.

Variables	MDTG group (*n* = 17)	CDTG group (*n* = 18)	*P*	*t*/χ^2^
Age (years)	58.2 ± 9.4	59.3 ± 8.3	0.730	-0.348
Disease duration (months)	8.1 ± 5.5	10.9 ± 4.6	0.099	-1.695
Height (cm)	167.7 ± 10.2	164.7 ± 5.1	0.278	1.112
Weight (kg)	70.2 ± 11.1	66.5 ± 8.8	0.287	1.082
BBS (score)	48.4 ± 1.6	48.3 ± 1.6	0.971	0.036
MMSE (score)	27.3 ± 3.1	28.4 ± 2.4	0.255	-1.158
Sex (male/female)	13/4	11/7	0.328	0.957
Stroke type (cerebral hemorrhage/cerebral infarction)	4/13	3/15	0.612	0.257
Affected side (left/right)	6/11	5/13	0.632	0.229

MDTG denotes the motor dual-task gait training; CDTG denotes the cognitive dual-task gait training.

This randomized controlled study was approved by the Chinese Clinical Trial Registry (registration number: ChiCTR2300077304) and the Ethics Committee of Beijing Sport University (approval number: 2023152H). Written informed consent was obtained from all participants prior to testing. This study adhered to CONSORT guidelines. All procedures performed in studies involving human participants were in accordance with the ethical standards of the institutional and/or national research committee and with the 1964 Helsinki declaration and its later amendments or comparable ethical standards.

### 2.2 Dual-task training protocol

Each of MDTG and CDTG comprised five training tasks. During each training session, participants completed all 5 tasks sequentially, with each task lasting 2 min and followed by a 1 min rest interval, constituting one training set. Two sets were performed per day, resulting in a total training duration of 30 min per session. Training sessions were conducted three times per week, with at least one rest day between sessions, over a period of 4 weeks. Both groups received dual-task training in addition to identical conventional rehabilitation. In addition to dual-task training, both groups received identical conventional rehabilitation, consisting of 30 min sessions, three times per week for 4 weeks. Conventional rehabilitation included active and passive range-of-motion exercises for the upper and lower limbs, muscle strengthening exercises, balance training, and gait training. After completing conventional rehabilitation and taking adequate rest, participants performed dual-task training. To ensure consistency in training intensity, frequency, and duration, all dual-task sessions were delivered under one-on-one supervision by the same therapist within each group.

During MDTG, participants walked on level ground at a self-selected speed while simultaneously performing the assigned motor task, with explicit instruction to maintain attention to both walking and the motor task. For all motor tasks, participants were instructed to perform the movements preferentially with the affected limb (hand or leg). If the affected limb was unable to fully execute the required movement, assistance from the unaffected limb was permitted to complete the task. Motor tasks were designed based on previous studies (Chen et al., 2026) and included: (1) object transfer task (participants used the unaffected hand to retrieve a lanyard from the pocket on the same side and transfer it to the affected hand, which then placed the lanyard around the neck); (2) object holding task (participants held a disposable cup filled with 450 mL of water in the affected hand, with the water surface maintained 1 cm below the rim, and were instructed to keep the cup steady without spilling); (3) coordination task (a ball was placed inside a net, which participants held with the unaffected hand while kicking the ball using the affected leg); (4) pick and place task (participants used the affected hand to retrieve a ball from the ipsilateral pocket, transfer it to the unaffected hand, and, after a 5s delay, return it to the pocket); (5) ball dribbling task (participants continuously bounced a ball on the floor using the affected hand).

During CDTG, participants walked on level ground at a self-selected speed while simultaneously performing the assigned cognitive task, with explicit instruction to maintain attention to both walking and the cognitive task. At the end of each week, the therapist reviewed participants’ performance during the week and adjusted the subsequent week’s cognitive training program based on their mastery of the cognitive tasks. Cognitive tasks were designed based on previous studies (Al-Yahya et al., 2011; Plummer et al., 2014; Chen et al., 2026) and included: (1) discrimination and decision-making task (participants were presented with a series of words whose meanings and font colors were incongruent, and were instructed to immediately verbalize either the color or the meaning of each word. Each set comprised 15 color words presented in a randomized order); (2) reaction time task (participants were randomly presented with a three-digit number between 100 and 999 and instructed to perform continuous subtraction by one); (3) working memory task (participants were first presented with a set of two character words and instructed to recall them in reverse order, with the task gradually increasing to three and four character word sets as proficiency improved); (4) verbal fluency task (participants were instructed to generate as many words as possible from a given character or to perform a word chain task based on a presented two character word); (5) information tracking task (participants were presented with a shopping list containing three to five items and instructed to recall them in order, with lists initially containing three items and gradually increasing to four and five items as proficiency improved).

### 2.3 Data collection

All participants underwent obstacle crossing gait and cortical activation tests at both pre- and post-training. For both tests, obstacle height was set at 10% leg-length of the affected limb, defined as the distance from the anterior superior iliac spine to the lateral malleolus. The obstacle consisted of a central wooden crossbar and two height-adjustable wooden support blocks, to enable adjustment of obstacle height. The crossbar was loosely placed on the support blocks so that it would fall off when touched by the foot, thereby minimizing the risk of tripping. Retroreflective markers were attached to both ends of the crossbar to enable identification of the obstacle’s position. Before testing, participants were explicitly instructed to perform obstacle crossing within their individual safety limits and to stop immediately if they perceived any potential danger. During testing, a therapist walked alongside the participant at a distance of approximately 1 m to prevent falls. Trials in which participants contacted the obstacle, lost balance, or required assistance from the therapist were considered unsuccessful and excluded from analysis. The pre-intervention data were collected from June to July 2023, and the post-intervention data were collected from July to August 2023.

#### 2.3.1 Obstacle crossing gait test

Before the gait test, participants wore tight-fitting clothing and 29 retroreflective markers were attached to their whole bodies according to the Helen Hayes protocol (Jeong et al., 2018). During testing, participants walked along an 8 m walkway at a self-selected speed, leading with the affected limb to cross the obstacle positioned at the center of the walkway. Marker positions and ground reaction forces during obstacle crossing were recorded synchronously using an 8-camera motion capture system (Oqus 700, Qualisys, Sweden, 200 Hz) and a force plate (Kistler 9286 B, Switzerland, 1000 Hz). A trial was considered valid when participants walked without adjusting their steps, the unaffected (trailing limb) foot fully contacted the force plate before the obstacle, and all retroreflective marker positions were successfully recorded. Each participant completed two valid trials, and the mean values were used for statistical analysis.

#### 2.3.2 Obstacle crossing cortical activation test

**Figure 2:**
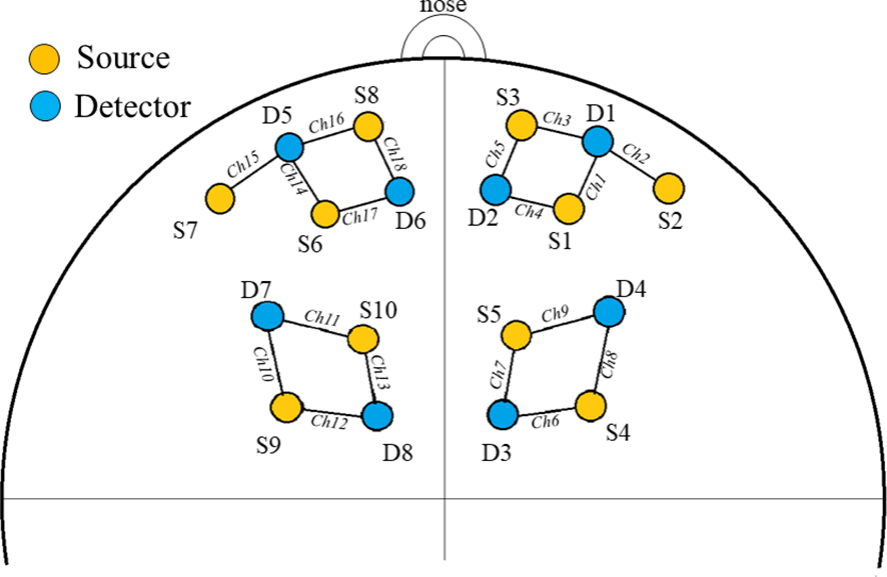
Schematic of fNIRS optode and channel placement.

**Figure 3:**
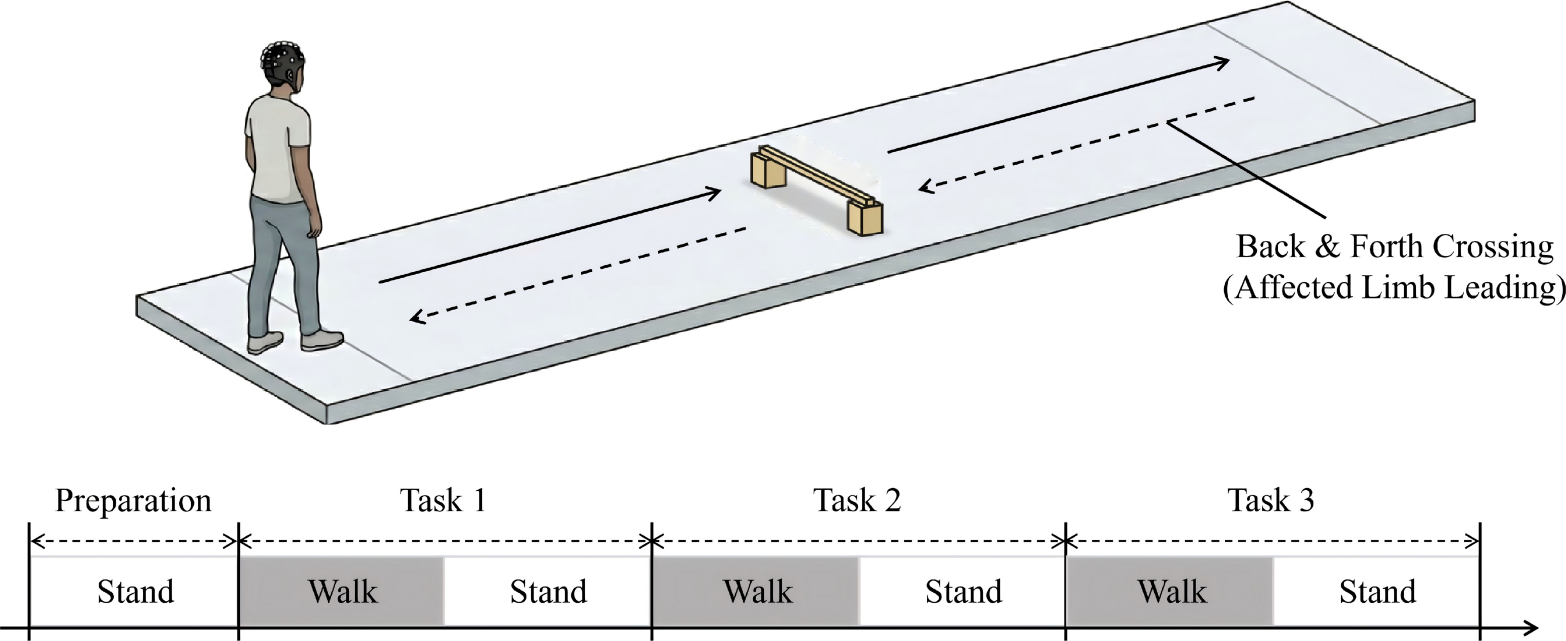
Flow diagram of obstacle crossing cortical activation test.

Before the cortical activation test, participants wore an fNIRS optode cap to position probes over the bilateral PFC and PMC. The optode arrangement followed the Artinis standard “4×4 + 2” template, with probe locations adhered to the international 10–20 system (Al-Yahya et al., 2016), resulting in the construction of 18 valid measurement channels. The specific optode arrangement and the corresponding cortical regions for each channel are shown in **Figure 2**. The cortical activation test consisted of a preparation phase and a task phase. During the preparation phase, participants stood still at the starting point of the walkway for 30 s to establish baseline signals. Upon receiving the experimenter’s “start” instruction, the task phase commenced. During this phase, participants walked at a self-selected speed, leading with the affected limb to repeatedly cross the obstacle positioned at the center of the walkway in a forth and back manner for a total duration of 30 s. Following the “stop” instruction, participants immediately ceased walking and maintained a stationary stance for 30 s. Upon receiving the “start” instruction again, participants commenced the next task phase. The task phase was performed three times in total for each cortical activation test (**Figure 3**). A fNIRS system (Artinis Brite MK III, Netherlands, 25Hz) was used to continuously record optical density signals from the bilateral PFC and PMC throughout the entire testing procedure. A trial was considered valid if participants completed uninterrupted walking and obstacle crossing without noticeable motion artifacts. The testing environment was kept quiet throughout, with instructions delivered solely by the experimenter.

### 2.4 Data processing

#### 2.4.1 Kinematic and kinetic data processing

All collected three-dimensional positions of the retroreflective markers were filtered with a low-pass Butterworth filter with a cut-off frequency of 13.3 Hz (Chen et al., 2024). Based on the marker positions of the heel, toe, and obstacle, spatio-temporal parameters such as step length, step width, and limb-obstacle distance were computed (Wan et al., 2025; Zhu et al., 2025). Whole-body marker positions were used to compute the position of the COM (Wan et al., 2025; Zhu et al., 2025). The center of pressure (COP) was derived from ground reaction forces and moments measured by the force plate (Jamshidi et al., 2010). Joint angles of the lower limbs were computed using the Euler angle method (Wan et al., 2025). Joint moments of the lower limbs were computed using the inverse dynamics method, with human inertial parameters determined according to the DeLeva’s modified Zatsiorsky-Seluyanov model (Wan et al., 2025). Joint moments were normalized to body weight and expressed in Nm/kg. Foot contact and toe-off were determined based on a vertical ground reaction force threshold of 25 N.

In this study, the limb that crossed the obstacle first was defined as the leading limb, while the limb that crossed subsequently was defined as the trailing limb (Zhu et al., 2025). An obstacle crossing gait cycle was defined as the interval from heel contact of the unaffected trailing limb before the obstacle to heel contact of the same limb after the obstacle, and was divided into four primary phases: (1) pre-obstacle double support phase, (2) swing phase of the leading limb, (3) mid-obstacle double support phase, and (4) swing phase of the trailing limb (Zhu et al., 2025). The duration of each phase was also expressed as a percentage of the total gait cycle.

Spatio-temporal parameters were also extracted from the obstacle crossing gait cycle, including crossing step length, crossing stride length, crossing step width, crossing gait speed, limb-obstacle distance, COM displacement, COM mean velocity, COP displacement, and COM-COP distance. Detailed definitions of these gait parameters are provided in **Table 2**. In addition, joint angles and moments analyzed in this study included: (1) the ranges of motion (ROM) of the hip, knee, and ankle joints of the leading limb during its swing phase; (2) three-dimensional hip joint angles and sagittal-plane knee and ankle angles of the swing limb at the moment when the leading limb’s toe was above the obstacle (T1) and when the trailing limb’s toe was above the obstacle (T2); (3) peak hip, knee, and ankle joint moments of the trailing limb during its stance phase; and (4) hip, knee, and ankle joint moments of the trailing limb at T1.

**Table 2:** Definition of spatio-temporal parameters.

Spatio-temporal parameters	Definition
Crossing step length (cm)	Anteroposterior distance between the heel marker of the trailing limb at initial contact before the obstacle and the heel marker of the leading limb at initial contact after the obstacle
Crossing stride length (cm)	Anteroposterior distance between the heel marker of the trailing limb at initial contact before the obstacle and the heel marker of the same trailing limb at initial contact after the obstacle
Crossing step width (cm)	Mediolateral distance between the heel marker of the trailing limb at initial contact before the obstacle and the heel marker of the leading limb at initial contact after the obstacle
Crossing gait speed (cm/s)	Crossing stride length divided by gait cycle duration
Toe-obstacle distance of the trailing limb (cm)	The minimum horizontal distance between the toe marker of the trailing limb and the obstacle before crossing
Toe-obstacle clearance of the leading limb (cm)	The vertical distance between the toe marker of the leading limb and the obstacle when the toe is above the obstacle during crossing
Toe-obstacle clearance of the trailing limb (cm)	The vertical distance between the toe marker of the trailing limb and the obstacle when the toe is above the obstacle during crossing
Heel-obstacle distance of the leading limb (cm)	The minimum horizontal distance between the heel marker of the leading limb and the obstacle after crossing
COM displacement (cm)	Maximum displacement of the COM projection on the support surface in the AP or ML direction
COM mean velocity (cm/s)	Mean velocity of the COM projection on the support surface in the AP or ML direction
COP displacement (cm)	Maximum displacement of the COP in the AP or ML direction
COM-COP distance (cm)	Maximum distance between the COM and COP in the AP or ML direction

COM denotes the center of mass; COP denotes the center of pressure; AP denotes the anteroposterior direction; ML denotes the mediolateral direction.

#### 2.4.2 Cortical activation data processing

In this study, cortical activation data were processed using the Homer2 toolbox (v2.8) in MATLAB (R2013b) (Huppert et al., 2009). Motion artifacts in the optical density signals were removed using a moving standard deviation method, with an artifact detection threshold set at 50 times the standard deviation of the preceding window. Both the moving standard deviation calculation window and the artifact correction window were 1 s in length. The optical density signals were filtered with a band-pass filter of 0.01~0.10 Hz to reduce noise arising from physiological fluctuations such as cardiac pulsation and respiration. Based on the modified Beer-Lambert law, changes in light attenuation were converted into oxyhemoglobin (HbO_2_) concentrations (Al-Yahya et al., 2016). For baseline correction, the HbO_2_ concentration during obstacle crossing was adjusted by subtracting the mean HbO_2_ concentration measured during the final 5 s of quiet standing prior to walking. Finally, the changes in oxygenated hemoglobin concentration (ΔHbO_2_) in the bilateral prefrontal cortex (reflecting attentional allocation and executive control during obstacle crossing) and premotor cortex (reflecting motor planning during obstacle crossing) were extracted as indices of cortical activation.

### 2.5 Statistical analysis

Statistical analyses were performed using IBM SPSS Statistics version 25.0. Categorical variables were expressed as frequencies, and intergroup comparisons were conducted using the *χ*^2^ test. Continuous variables were assessed for normality using the Shapiro-Wilk test; data following a normal distribution were expressed as mean ± standard deviation, and the homogeneity of variances between groups was assessed using Levene’s test. A 2 × 2 mixed-design two-way repeated measures analysis of variance (ANOVA) was performed to examine the effects of group (CDTG vs. MDTG, between-subject factor) and time (pre-training vs. post-4-week training, within-subject factor) on each outcome measure. The Bonferroni correction was applied for multiple comparisons to control the Type I error rate. Effect sizes were expressed as η^2^_p_, and the mean difference (MD) between pre- and post-training values was calculated for each outcome measure. Only participants who completed all interventions and assessments were included in the final statistical analysis. Statistical significance was defined as the probability of a Type I error not exceeding 0.05.

## 3 Results

### 3.1 Pre- and post-training comparison of gait characteristics between groups

ANOVA revealed no significant interaction between time and group for gait spatio-temporal parameters during obstacle crossing (*P* > 0.05) (**Table 3**). A significant main effect of time was observed for crossing stride length (F = 10.799, MD = 6.5 cm, 95%CI: 2.5 to 10.5 cm), crossing step length (F = 4.618, MD = 2.0 cm, 95%CI: 0.1 to 4.0 cm), crossing step width (F = 6.109, MD = 1.3 cm, 95%CI: 0.2 to 2.3 cm), crossing gait speed (F = 8.039, MD = 5.6 cm/s, 95%CI: 1.6 to 9.7 cm/s), toe-obstacle clearance of the leading limb (F = 17.433, MD = 1.8 cm, 95%CI: 0.9 to 2.7 cm), toe-obstacle distance of the trailing limb (F = 13.241, MD = 3.1 cm, 95%CI: 1.4 to 4.8 cm), and toe-obstacle clearance of the trailing limb (F = 11.834, MD = 2.1 cm, 95%CI: 0.9 to 3.4 cm). All of these parameters were greater post-training compared with pre-training. No significant main effect of group was observed for any gait spatio-temporal parameter during obstacle crossing (*P* > 0.05).

**Table 3:** Pre- and post-training comparison of gait spatio-temporal parameters between groups.

Gait spatio-temporal parameters	MDTG group (*n* = 17)		CDTG group (*n* = 18)		Main effect of time		Main effect of group	
	Pre-training	Post-training	Pre-training	Post-training	*P*	η^2^_p_	*P*	η^2^_p_
Crossing stride length (cm)	98.1 ± 23.4	103.6 ± 25.1	88.6 ± 23.7	96.1 ± 25.1	0.003^*^	0.265	0.313	0.034
Crossing step length (cm)	53.3 ± 8.1	55.3 ± 10.2	49.3 ± 7.7	51.4 ± 8.8	0.040^*^	0.133	0.190	0.057
Crossing step width (cm)	15.8 ± 3.7	17.5 ± 3.6	16.6 ± 4.2	17.3 ± 3.0	0.019^*^	0.169	0.803	0.002
Crossing gait speed (cm/s)	56.5 ± 20.9	62.3 ± 22.3	51.9 ± 23.0	57.4 ± 22.9	0.008^*^	0.211	0.539	0.013
pre-obstacle double support phase (%)	16.2 ± 10.1	15.7 ± 7.9	16.2 ± 10.6	15.0 ± 7.5	0.523	0.014	0.908	0.001
mid-obstacle double support phase (%)	11.2 ± 5.2	11.9 ± 7.0	13.8 ± 7.9	12.9 ± 8.9	0.871	0.001	0.476	0.017
swing phase of the leading limb (%)	41.2 ± 8.7	41.7 ± 7.6	40.9 ± 9.2	41.7 ± 7.2	0.496	0.016	0.977	0.001
swing phase of the trailing limb (%)	31.5 ± 8.9	30.8 ± 8.7	29.1 ± 10.0	30.4 ± 8.4	0.707	0.005	0.656	0.007
Toe-obstacle clearance of the leading limb (cm)	13.4 ± 5.0	15.0 ± 5.0	13.1 ± 3.4	15.1 ± 4.5	0.001^*^	0.368	0.950	0.001
Heel-obstacle distance of the leading limb (cm)	14.3 ± 5.4	13.6 ± 5.5	13.1 ± 4.6	13.0 ± 5.9	0.585	0.010	0.621	0.008
Toe-obstacle distance of the trailing limb (cm)	12.9 ± 5.0	16.0 ± 7.5	10.4 ± 4.2	13.4 ± 4.3	0.001^*^	0.306	0.143	0.070
Toe-obstacle clearance of the trailing limb (cm)	13.6 ± 6.6	15.1 ± 8.1	13.4 ± 5.4	16.2 ± 6.3	0.002^*^	0.283	0.839	0.001

MDTG denotes the motor dual-task gait training; CDTG denotes the cognitive dual-task gait training; * indicates a statistically significant difference between pre- and post-training values.

ANOVA revealed no significant interaction between time and group for lower limb joint angles during obstacle crossing (*P* > 0.05) (**Table 4**). A significant main effect of time was observed for hip flexion angle of the leading limb at T1 (F = 5.818, MD = 2.5°, 95%CI: 0.4 to 4.7°) and knee flexion angle of the trailing limb at T2 (F = 15.156, MD = 4.6°, 95%CI: 2.2 to 7.1°). All of these lower limb joint angles were greater post-training compared with pre-training. No significant main effect of group was observed for any lower limb joint angle during obstacle crossing (*P* > 0.05).

**Table 4:** Pre- and post-training comparison of lower limb joint angles between groups.

Joint angles (°)	MDTG group (*n* = 17)		CDTG group (*n* = 18)		Main effect of time		Main effect of group	
	Pre-training	Post-training	Pre-training	Post-training	*P*	η^2^_p_	*P*	η^2^_p_
Hip flexion-extension ROM of the leading limb	44.2 ± 8.5	46.0 ± 9.5	43.5 ± 9.4	44.3 ± 9.6	0.361	0.028	0.687	0.005
Knee flexion-extension ROM of the leading limb	60.1 ± 17.8	62.3 ± 19.3	61.8 ± 12.9	62.3 ± 13.9	0.328	0.032	0.878	0.001
Ankle flexion-extension ROM of the leading limb	17.5 ± 7.9	17.6 ± 5.4	18.1 ± 7.0	19.2 ± 7.1	0.627	0.008	0.608	0.009
Hip flexion angle of the leading limb at T1	70.3 ± 8.8	73.1 ± 8.7	70.2 ± 9.5	72.4 ± 9.5	0.022^*^	0.162	0.887	0.001
Hip abduction angle of the leading limb at T1	4.2 ± 4.4	5.6 ± 4.8	6.4 ± 4.2	6.6 ± 4.0	0.288	0.038	0.257	0.043
Hip extorsion angle of the leading limb at T1	6.2 ± 7.2	8.2 ± 9.0	3.5 ± 11.7	2.1 ± 9.9	0.804	0.002	0.175	0.060
Knee flexion angle of the leading limb at T1	73.5 ± 15.4	72.0 ± 11.9	77.3 ± 12.1	78.7 ± 12.0	0.995	0.001	0.235	0.047
Ankle plantarflexion angle of the leading limb at T1	3.1 ± 5.5	4.0 ± 4.9	1.7 ± 5.1	0.7 ± 4.8	0.937	0.001	0.178	0.060
Hip flexion angle of the trailing limb at T2	48.0 ± 11.1	50.2 ± 9.3	51.9 ± 10.8	53.3 ± 8.6	0.194	0.056	0.290	0.037
Hip abduction angle of the trailing limb at T2	7.8 ± 5.3	7.9 ± 5.1	9.1 ± 5.9	10.0 ± 4.4	0.475	0.017	0.329	0.032
Hip intorsion angle of the trailing limb at T2	2.4 ± 8.3	2.4 ± 7.0	7.6 ± 6.9	4.8 ± 6.4	0.197	0.055	0.107	0.084
Knee flexion angle of the trailing limb at T2	96.2 ± 10.2	99.6 ± 11.0	97.7 ± 8.8	103.5 ± 9.9	0.001^*^	0.336	0.427	0.021
Ankle plantarflexion angle of the trailing limb at T2	7.6 ± 14.0	8.8 ± 13.6	10.4 ± 12.0	10.1 ± 11.9	0.662	0.006	0.655	0.007

MDTG denotes the motor dual-task gait training; CDTG denotes the cognitive dual-task gait training; T1 denotes the moment when the leading limb’s toe was above the obstacle; T2 denotes the moment when the trailing limb’s toe was above the obstacle; ROM denotes the ranges of motion; * indicates a statistically significant difference between pre- and post-training values.

ANOVA revealed no significant interaction between time and group for joint moments of the trailing limb during obstacle crossing (*P* > 0.05) (**Table 5**). A significant main effect of time was observed for peak hip flexion moment of the trailing limb (F = 5.520, MD = 0.10 Nm/kg, 95% CI: 0.0 to 0.1 Nm/kg). This joint moment was greater post-training compared with pre-training. No significant main effect of group was observed for any joint moment of the trailing limb during obstacle crossing (*P* > 0.05).

**Table 5:** Pre- and post-training comparison of lower limb joint moments of the trailing limb between groups.

Joint moments of the trailing limb (Nm/kg)	MDTG group (*n* = 17)		CDTG group (*n* = 18)		Main effect of time		Main effect of group	
	Pre-training	Post-training	Pre-training	Post-training	*P*	η^2^_p_	*P*	η^2^_p_
Hip extension moment at T1	0.26 ± 0.27	0.25 ± 0.27	0.32 ± 0.23	0.25 ± 0.19	0.188	0.057	0.715	0.004
Knee flexion (+)/extension (-) moment at T1	-0.03 ± 0.28	-0.04 ± 0.18	0.02 ± 0.21	-0.02 ± 0.23	0.457	0.019	0.683	0.006
Ankle plantarflexion moment at T1	0.51 ± 0.21	0.44 ± 0.19	0.52 ± 0.19	0.51 ± 0.18	0.164	0.064	0.466	0.018
Peak hip extension moment	0.65 ± 0.24	0.66 ± 0.28	0.60 ± 0.21	0.61 ± 0.16	0.915	0.001	0.503	0.015
Peak hip flexion moment	0.15 ± 0.20	0.23 ± 0.18	0.14 ± 0.23	0.19 ± 0.27	0.026^*^	0.155	0.785	0.003
Peak knee flexion moment	0.10 ± 0.23	0.14 ± 0.18	0.09 ± 0.19	0.07 ± 0.22	0.671	0.006	0.598	0.009
Peak knee extension moment	0.47 ± 0.28	0.47 ± 0.19	0.37 ± 0.33	0.41 ± 0.35	0.467	0.018	0.424	0.021
Peak ankle plantarflexion moment	1.19 ± 0.21	1.19 ± 0.19	1.07 ± 0.26	1.12 ± 0.26	0.209	0.052	0.212	0.051
Minimum ankle plantarflexion moment	0.16 ± 0.14	0.15 ± 0.16	0.23 ± 0.17	0.22 ± 0.16	0.694	0.005	0.171	0.062

MDTG denotes the motor dual-task gait training; CDTG denotes the cognitive dual-task gait training; T1 denotes the moment when the leading limb’s toe was above the obstacle; * indicates a statistically significant difference between pre- and post-training values.

ANOVA revealed no significant interaction between time and group for postural stability parameters during obstacle crossing (*P* > 0.05) (**Table 6**). A significant main effect of time was observed for anteroposterior (AP) COM displacement (F = 5.414, MD = 3.7 cm, 95% CI: 0.5 to 7.0 cm), mediolateral (ML) COM displacement (F = 4.855, MD = 0.3 cm, 95% CI: 0.0 to 0.7 cm), AP COM mean velocity (F = 4.829, MD = 5.5 cm/s, 95% CI: 0.4 ~ 10.6), and AP COM-COP distance (F = 7.992, MD = 1.5 cm, 95% CI: 0.4 to 2.6 cm). All of these postural stability parameters were greater post-training compared with pre-training. No significant main effect of group was observed for any postural stability parameter during obstacle crossing (*P* > 0.05).

**Table 6:** Pre- and post-training comparison of postural stability parameters between groups.

Postural stability parameters	MDTG group (*n* = 17)		CDTG group (*n* = 18)		Main effect of time		Main effect of group	
	Pre-training	Post-training	Pre-training	Post-training	*P*	η^2^_p_	*P*	η^2^_p_
AP COM displacement (cm)	35.7 ± 10.3	39.3 ± 13.2	31.2 ± 13.4	34.9 ± 12.7	0.027^*^	0.153	0.286	0.038
ML COM displacement (cm)	3.1 ± 1.2	3.4 ± 1.1	3.3 ± 1.6	3.8 ± 1.8	0.035^*^	0.139	0.536	0.013
AP COM mean velocity (cm/s)	66.5 ± 20.0	72.1 ± 22.8	62.0 ± 22.0	67.3 ± 24.8	0.036^*^	0.139	0.543	0.012
ML COM mean velocity (cm/s)	17.4 ± 4.9	18.6 ± 5.3	17.3 ± 5.2	18.1 ± 6.2	0.154	0.067	0.860	0.001
AP COP displacement (cm)	9.9 ± 2.5	10.1 ± 2.9	8.3 ± 2.9	8.9 ± 3.0	0.304	0.035	0.142	0.070
ML COP displacement (cm)	2.3 ± 1.2	2.3 ± 0.9	1.9 ± 0.8	2.3 ± 1.1	0.662	0.006	0.299	0.036
AP COM-COP distance (cm)	17.0 ± 4.6	18.5 ± 5.0	16.2 ± 5.1	17.8 ± 5.6	0.008^*^	0.210	0.662	0.006
ML COM-COP distance (cm)	9.3 ± 2.1	9.9 ± 2.9	8.9 ± 2.8	9.4 ± 2.7	0.143	0.070	0.636	0.008

MDTG denotes the motor dual-task gait training; CDTG denotes the cognitive dual-task gait training; COM denotes the center of mass; COP denotes the center of pressure; AP denotes the anteroposterior direction; ML denotes the mediolateral direction; * indicates a statistically significant difference between pre- and post-training values.

### 3.2 Pre- and post-training comparison of cortical activation between groups

ANOVA revealed no significant interaction between time and group for cortical activation during obstacle crossing (*P* > 0.05) (**Table 7**). A significant main effect of time was observed for ΔHbO_2_ in the affected PFC (F = 4.229, MD = -0.23 μmol/L, 95% CI: -0.45 to 0 μmol/L) and the unaffected PFC (F = 5.50, MD = -0.32 μmol/L, 95% CI: -0.60 to -0.04 μmol/L). All of these cortical activation were lower post-training compared with pre-training. No significant main effect of group was observed for any cortical activation during obstacle crossing (*P* > 0.05).

**Table 7:** Pre- and post-training comparison of cortical activation between groups.

ΔHbO_2_ (μmol/L)	MDTG group (*n* = 17)		CDTG group (*n* = 18)		Main effect of time		Main effect of group	
	Pre-training	Post-training	Pre-training	Post-training	*P*	η^2^_p_	*P*	η^2^_p_
Affected PFC	1.36 ± 0.63	1.28 ± 0.71	1.37 ± 0.74	1.00 ± 0.50	0.049*	0.131	0.526	0.015
Unaffected PFC	1.50 ± 0.55	1.21 ± 0.44	1.46 ± 0.89	1.10 ± 0.82	0.027*	0.169	0.740	0.004
Affected PMC	0.68 ± 0.4	0.71 ± 0.44	0.74 ± 0.45	0.54 ± 0.24	0.231	0.055	0.705	0.006
Unaffected PMC	0.66 ± 0.49	0.75 ± 0.32	0.72 ± 0.37	0.60 ± 0.39	0.930	0.001	0.722	0.005

MDTG denotes the motor dual-task gait training; CDTG denotes the cognitive dual-task gait training; PFC denotes the prefrontal cortex; PMC denotes the premotor cortex; * indicates a statistically significant difference between pre- and post-training values.

## 4 Discussion

Due to impairments in musculoskeletal function, attention, spatial perception, and proprioception, stroke patients often exhibit abnormal gait patterns during obstacle crossing, which increases their risk of falling (Den Otter et al., 2005; Sparrow and Tirosh, 2005; Wan et al., 2025). In this study, stroke patients underwent 4 weeks of either MDTG or CDTG. Both trainings led to improvements in obstacle crossing gait and cortical activation compared with pre-training levels, potentially contributing to a reduced risk of falling. Specifically, participants demonstrated improved postural stability, enhanced control of the swing limb and COM, and increased efficiency in cortical resource utilization during obstacle crossing. However, no significant differences were observed between the two training modalities in terms of functional improvements.

Gait speed, step length, and step width are closely linked to lower limb muscle strength and postural control, serving as key parameters for assessing walking stability in stroke patients (Patterson et al., 2007; Hak et al., 2013, Hak et al., 2015; Feld, 2020; Wang and Bhatt, 2022). In this study, following 4 weeks of dual-task training, participants in both groups demonstrated increased crossing gait speed, extended crossing stride and step lengths, and widened crossing step width during obstacle crossing. These findings support Hypothesis 1, suggesting improved dynamic postural stability in the patients. A prospective study by Feld *et al*. (Feld, 2020) reported that, compared with fallers, stroke non-fallers exhibited faster gait speed and longer stride length during obstacle crossing, reflecting greater balance ability and lower limb strength, which enabled more effective gait adjustments. Den et al. (Den Otter et al., 2005) found that patients who successfully crossed obstacles tended to adopt a strategy of increasing step length. Longer crossing stride and step lengths indicate greater displacement per step, which not only expands the base of support at each phase of obstacle crossing but also helps maintain gait continuity, facilitating balance when facing perturbations or shifts of the COM (Hak et al., 2015). Other studies (Hak et al., 2013; Kao et al., 2014) have shown that increased step width during walking enlarges the lateral base of support, enhancing mediolateral stability in stroke patients. Therefore, the observed post-training improvements in gait speed, step length, and step width in the present study suggest that both MDTG and CDTG can enhance postural control and balance during obstacle crossing, allowing patients to complete the task more efficiently while maintaining stability, thereby reducing potential fall risk.

The limb-obstacle distance directly reflects the relative position between the limb and the obstacle, and is closely associated with control of the swing limb during obstacle crossing in stroke patients (van Swigchem et al., 2013; Chen et al., 2019; Hösl et al., 2019). Appropriately increasing limb-obstacle distance provides patients with adequate time and space to adjust the trajectory of the swing limb, facilitating successful clearance and reducing the risk of foot contact with obstacle (Chen et al., 2015). Wan et al (Wan et al., 2025). developed a fall risk prediction model for stroke patients and reported that increasing limb-obstacle distance during obstacle crossing helps prevent falls. Specifically, for each unit increase in toe-obstacle clearance of the trailing limb, the risk of falling decreased by 18.9%. Toe-obstacle clearance is primarily determined by the flexion angles of the hip, knee, and ankle joints of the swing limb (Said et al., 2005). Wan et al (Wan et al., 2025). further noted that reduced toe-obstacle clearance of the swing limb during obstacle crossing results from impaired control of the swing limb, mainly due to restricted hip and knee flexion. Consistently, a retrospective study by Benson et al (Benson et al., 2018). indicated that limited knee motion during obstacle crossing may signal an elevated risk of tripping. In this study, after 4 weeks of dual-task training, participants in both groups exhibited increased limb-obstacle clearance during obstacle crossing, including greater toe-obstacle clearance of the leading limb, toe-obstacle clearance of the trailing limb, and toe-obstacle distance of the trailing limb compared with pre-training values. In addition, hip flexion angle of the leading limb at T1 and knee flexion angle of the trailing limb at T2 were significantly increased. These improvements in gait parameters support Hypothesis 1, indicating that dual-task training effectively optimizes the movement of the hip joint in the affected leading limb and the knee joint in the unaffected trailing limb, thereby enhancing patients’ ability to control the spatial relationship between their limbs and the obstacle. By increasing joint angles and optimizing lower limb coordination patterns, patients are able to allocate greater space for foot trajectory adjustments during obstacle crossing. This, in turn, increases limb-obstacle distance, facilitates successful crossing, and reduces the risk of falls caused by foot contact with obstacle.

This study further demonstrated that, following 4 weeks of dual-task training, both groups exhibited significant increases in AP and ML COM displacement, ML COM mean velocity, AP COM-COP distance during obstacle crossing, as well as peak hip flexion moment of the trailing limb during its stance phase, compared with pre-training values. These findings support Hypothesis 1. COM velocity during obstacle crossing reflects dynamic postural stability, and changes in COM velocity are closely associated with fall risk (Studenski et al., 2003; Verghese et al., 2009). Previous study (Judge et al., 1996) has shown that a 0.1 m/s decrease in walking COM velocity corresponds to an approximately 10 % decline in physical performance. In this study, participants exhibited a significant increase in ML COM mean velocity following training, suggesting that dual-task training can effectively enhance postural control in stroke patients. Previous study (Chen et al., 2019) has shown that, compared with healthy individuals, stroke patients exhibit reduced AP COM velocity during obstacle crossing, providing additional time to adjust the trajectory of the swing limb and thereby mitigating the risk of forward falls. Said et al (Said et al., 2008). further reported that stroke patients’ COM remains closer to the COP during obstacle crossing, effectively shortening the resisted arm of the stance limb. These gait adjustments represent a conservative strategy adopted to maintain postural stability in stroke patients. In this study, following dual-task training, participants demonstrated increased gait speed, greater COM displacement and velocity, expanded COM-COP distance, and enhanced peak hip flexion moment of the trailing limb. These findings suggest that dual-task training enables stroke patients to rely more on the hip strength of the unaffected stance limb to maintain core stability, improving the control of COM and allowing obstacle crossing without solely depending on conservative strategies to maintain balance.

The PFC plays a pivotal role in the execution of cognitive functions in stroke patients, particularly in processes such as attentional control, decision making, and task management (Miller and Cohen, 2001). Evidence indicates that the PFC becomes highly activated in individuals during obstacle crossing, mobilizing more cognitive resources to coordinate gait control (Chen, 2022). However, in stroke patients, excessive activation of the PFC during movement is generally interpreted as a compensatory mechanism intended to offset limitations in cognitive resource regulation efficiency (Mihara et al., 2007; Hawkins et al., 2018; Mori et al., 2018). This study revealed that, following 4 weeks of dual-task training, patients in both groups exhibited significantly reduced activation of both the affected and unaffected PFC during obstacle crossing, partially supporting Hypothesis 2. This reduction is likely attributable to improved balance control brought about by training, which rendered patients’ obstacle crossing movements more proficient and thereby reduced their reliance on cognitive resources during task execution. Meester et al. (Meester, 2016) reported that reduced PFC activation reflects decreased cognitive load and improved task performance efficiency under dual-task conditions. Therefore, the reduced activation in bilateral PFC during obstacle crossing post-training indicates that both dual-task training programs promote the automation of obstacle crossing movements, result in more efficient PFC processing of the task, and reduce patients’ reliance on cognitive resources, thereby achieving economization of cerebral resources. In addition, the PMC plays a critical role in motor planning and execution in stroke patients, primarily responsible for integrating sensory information, generating motor plans, and transmitting commands to the motor cortex (Johansen-Berg and Matthews, 2002; Kantak et al., 2012). In this study, following 4 weeks of training, no significant changes were observed in bilateral PMC activation during obstacle crossing in either group. This finding further suggests that both dual-task training programs may primarily enhance the allocation and integration of neural resources, improving obstacle crossing performance by increasing the efficiency of cognitive resource utilization, rather than directly modifying motor planning mechanisms.

The findings of this study further indicate that, following 4 weeks of dual-task training, no significant time × group interaction effects were observed for any of the gait or cortical activation parameters during obstacle crossing in stroke patients, failing to support Hypothesis 3. These findings suggest that both training programs produce similar effects in enhancing postural stability, control of the swing limb and COM, and efficiency of cerebral resource utilization during obstacle crossing. In this study, both MDTG and CDTG adopted similar training duration and intensity, and provided walking attention regulation training to the participants, which effectively improved the neuromuscular control, gait stability, and the efficiency of cerebral resource utilization in stroke patients navigating complex environments. In addition, although the two paradigms differ in task structure, both require participants to handle divided-attention demands during walking, which may activate a common neuroplastic pathway and enhance prefrontal cortex efficiency, and this could be an important reason for the similar improvements observed between the two training modalities. Meanwhile, when training load is comparable, differences in training type may have a relatively limited impact on obstacle crossing ability. Future studies could investigate potential differences between MDTG and CDTG in enhancing obstacle crossing performance and preventing falls in stroke patients by increasing training intensity or extending the training duration.

### 4.1 Limitations and future directions

Functional recovery in stroke patients is influenced by multiple factors, including lesion location, lesion lateralization (left or right hemisphere), severity of injury, rehabilitation duration, and individual baseline physical capacity. Inter-individual differences may have partially influenced the between-group differences in gait performance and cortical activation during obstacle crossing. Therefore, further studies should consider the potential influence of these factors on the effect of rehabilitation training when grouping participants and selecting independent variables. Similarly, rehabilitation practice should adopt more individualized intervention strategies to optimize training efficacy. In addition, the inclusion of a conventional rehabilitation control group in future studies would help to further enhance the internal validity of the research and more clearly distinguish the training-specific effects from those of spontaneous recovery and practice effects. Moreover, this study focused on changes in the amplitude of ΔHbO_2_ and did not examine brain-behavior correlations. Future studies are warranted to explore the associations between cortical activation and gait performance to further elucidate the neural mechanisms underlying dual-task training. Furthermore, this study did not report failure-related outcomes such as obstacle-contact rate and loss-of-balance frequency, which may limit a comprehensive assessment of fall risk during obstacle crossing. Future studies are warranted to incorporate these outcomes to more comprehensively evaluate the impact of dual-task training on real-world fall risk in stroke patients. Finally, the lack of significant between-group differences observed in this study may, in addition to similar training duration and intensity, be related to the relatively small sample size, limited statistical power, and relatively short intervention period, which may have constrained the ability to detect group × time interaction effects. Therefore, future studies are encouraged to increase sample size, extend intervention duration, and further optimize experimental design to more sensitively detect potential differences between different dual-task training modalities.

## 5 Conclusion

A 4-week program of either MDTG or CDTG effectively improved postural stability, enhanced control of the swing limb and COM, and increased efficiency in cortical resource utilization during obstacle crossing in stroke patients. However, no differences were observed between the two training modalities in their effects on gait or cortical activation during obstacle crossing. Therefore, in the rehabilitation of obstacle-crossing ability for stroke patients, both training programs can be employed to improve performance and reduce potential fall risk.

## Data Availability

The raw data supporting the conclusions of this article will be made available by the authors, without undue reservation.
